# Granulomatosis With Polyangiitis: Remissions in a Thiopurine Fast Metabolizer Over 20 Years

**DOI:** 10.7759/cureus.12616

**Published:** 2021-01-11

**Authors:** Ana Pedroso, Lutz Beckert

**Affiliations:** 1 Internal Medicine, Hospital São Francisco Xavier, Lisboa, PRT; 2 Respiratory Medicine, Canterbury District Health Board, Christchurch, NZL

**Keywords:** azathioprine, allopurinol, granulomatosis, polyangiitis, thiopurines

## Abstract

A 35-year-old Maori man presented with lethargy, nasal congestion, scleritis, epistaxis, progressive shortness of breath, myalgia, and pleuritic pain. His CT scan showed bilateral pulmonary infiltrates and a corneal biopsy confirmed granulomatosis with polyangiitis (GPA).

Remission was achieved with cyclophosphamide-based regimen. Maintaining remission proved difficult because of treatment side effects or relapses on cyclophosphamide, methotrexate, or azathioprine maintenance regimes. Overall, he needed six courses of pulse cyclophosphamide / IV methylprednisolone therapy in his first 10 years of treatment.

He was identified to be an azathioprine fast metabolizer producing little active metabolite at usual doses. The combination of low dose azathioprine / allopurinol achieved therapeutic level of 6-thioguanine nucleotides (TGNs) to achieve long-term remission. Low dose azathioprine / allopurinol at levels to control inflammatory bowel disease achieve long-term remission of GPA. He has been in remission for 10 years without side effects or relapses.

## Introduction

Granulomatosis with polyangiitis (GPA) is a multisystem disorder characterized by necrotizing granulomatous inflammation, which affects small- and medium-sized blood vessels [1].

Patients present with upper airway symptoms, such as nasal discharge, sinusitis, or epistaxis and septum destruction can occur, causing saddle nose deformity. Disease progression evolves conferring constitutional symptoms as well as arthralgias, cutaneous vasculitis, mononeuritis or polyneuritis and renal involvement. In 8%-16% of patients, ocular manifestations may be the initial presentation [2].

Diagnosis of GPA is achieved through clinical assessment, serological tests for anti-neutrophil cytoplasmic antibodies (ANCA), and histological analysis. The American College of Rheumatology defined GPA by the presence of at least two of the following: 1) sinus involvement; 2) radiology showing lung nodules, fixed pulmonary infiltrates or cavities; 3) urinary sediment with hematuria or red cell casts; and 4) histological granulomas within an artery or in the perivascular area of an artery or arteriole [3].

New Zealand has a high prevalence of GPA 2-150/1,000,000 population, which is part of a latitude-dependent incidence gradient [4]. GPA can occur in all ethnic groups and affect equally both genders, with a mean age at diagnosis of 40 years [5].

Previously fatal, 30%-93% of patients achieve remission with the use of immunosuppressive drugs and corticosteroids, however, relapses remain frequent (18%-40% at two years) and maintenance treatment is a therapeutic challenge [5].

Azathioprine undergoes extensive metabolism in vivo causing large therapeutic variability. The intracellular metabolites 6-thioguanine nucleotides (TGNs) are responsible for most of its immunosuppressive and myelotoxic effects [6]. The enzyme thiopurine methyltransferase which metabolizes azathioprine has a vast polymorphism [6], causing different outcomes in the patients receiving thiopurines and making azathioprine at time clinically difficult to use.

## Case presentation

**Figure 1:**
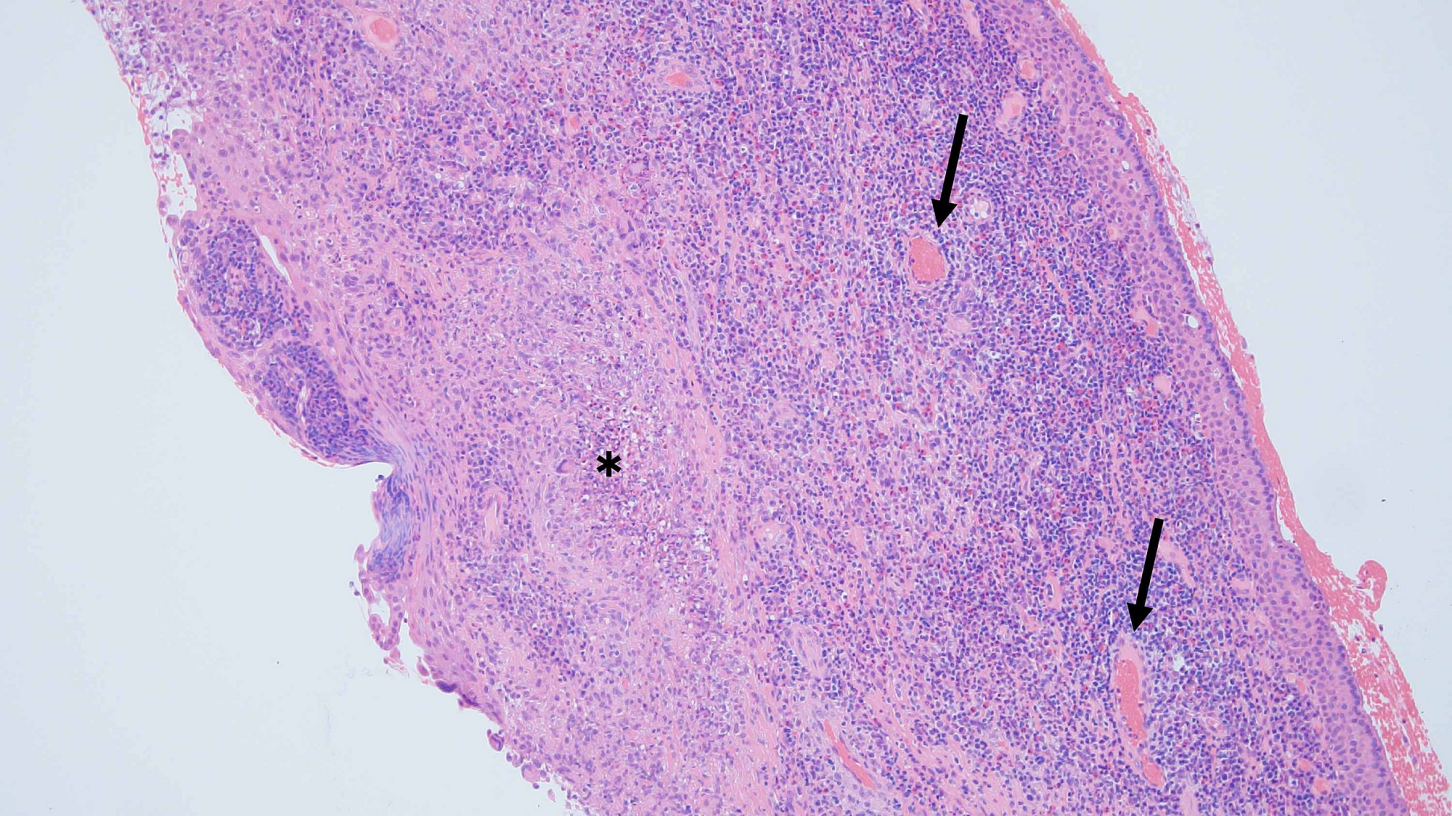
Histopathology showing corneal mucosa with inflammation and necrotising granuloma (*) and perivenular inflammation (arrows), (hematoxylin and eosin; original magnification: 100x).

In 1999 a 35-year-old Maori man presented with five weeks of lethargy, nasal congestion, scleritis, epistaxis, progressive shortness of breath, myalgia, and pleuritic pain. His CT scan showed bilateral pulmonary infiltrates and a corneal biopsy supported the GPA diagnosis (Figure 1).

During the first five years of his disease remission was achieved with oral and later pulse cyclophosphamide / methylprednisolone therapy. On cyclophosphamide maintenance therapy he suffered the side effects of hemorrhagic cystitis, bone marrow suppression, and varicella zoster. On low dose maintenance doses of cyclophosphamide or methotrexate he suffered relapses, all were successfully treated with pulse cyclophosphamide / methylprednisolone therapy.

**Figure 2:**
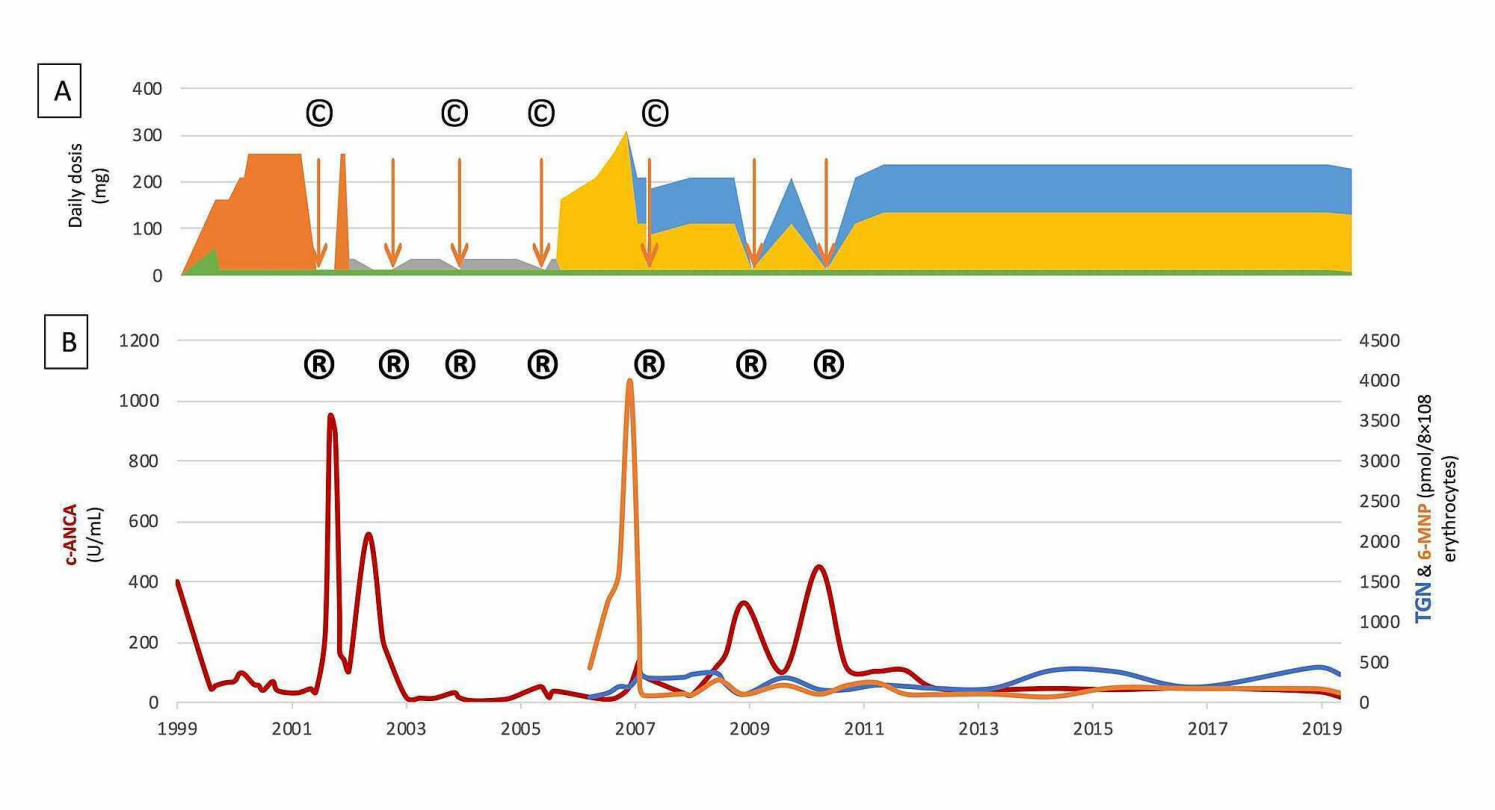
A) Treatment regimes and their complications. B) Relation between azathioprine metabolites (active TGN and nonactive 6-MMP) and disease activity (c-ANCA and relapses), across disease history. A) Treatment regimens: green - prednisolone, orange - cyclophosphamide, gray - methotrexate, yellow - azathioprine, blue - allopurinol. Arrows are cyclophosphamide pulses. © - treatment complications. B) Azathioprine metabolites: blue - active TGN, orange - nonactive 6-MMP. Disease activity shown by c-ANCA, in red, and relapses - ®. TGN, 6-thioguanine nucleotides; 6-MMP, 6-methylmercaptopurine; c-ANCA, cytoplasm anti-neutrophil cytoplasmic antibodies

After five years of trying to prevent relapses, azathioprine was tried. However, despite increasing his azathioprine dose to 300 mg once daily he suffered further relapses. His active nucleotide levels, TGN, only rose to 201 pmol/8×108 erythrocytes, while his thiopurine metabolite, 6-methylmercaptopurine (6-MMP), increased from 1617 to 3994 pmol/L (Figure 2). This suggested that his enzyme system was shunting into the 6-MMP pathway. This was confirmed by detecting a high thiopurine methyl transferase of 18.1 units/mL erythrocytes. 

Instead of increasing the azathioprine dose, allopurinol 100 mg/day was added to stop shunting into the 6-MMP pathway. Initially, aiming for an azathioprine active metabolize range for solid organ transplant did not provide enough immunosuppression [6-7]. A slide increase of the dose to reach the therapeutic targets suggested to treat inflammatory bowel disease [8-9], with an active TGN level above 235 pmol/8x108 erythrocytes, finally controlled his GPA.

Overall, he needed six courses of pulse cyclophosphamide / methylprednisolone therapy during his first 10 years of treatment, suffered treatment-related sided effect and was on oral prednisone. On his second 10 years of treatment with azathioprine 125 mg / allopurinol 100 mg he was able to be weaned off prednisone, he had no evidence of clinical, laboratorial or radiological relapses, maintained normal lung function, and continued to work full time.

## Discussion

Pulse therapy with cyclophosphamide / methylprednisolone has dramatically improved the survival of GPA, however, maintaining long-term remission is still a therapeutic challenge. Remission can be maintained with cyclophosphamide, methotrexate or azathioprine, but this therapy comes with significant toxicity and an approximately 70%-80% success rate. Low dose rituximab therapy has been suggested a long-term immunosuppressive regime, however, it is expensive and comes with higher risk of infection [10].

The case presented is typical for the ease of introducing remissions, the associated toxicities and the difficulties maintaining long-term remissions. By identifying the shunt of azathioprine metabolites and blocking this pathway with allopurinol, we were able to achieve long-term immunosuppression at a therapeutic active nucleotide range used to control inflammatory bowel disease [8-9].

## Conclusions

This case shows that long-term remission can be achieved with a cheap regime of azathioprine / allopurinol and by monitoring the active metabolites. After 10 years of treatment and prednisone weaning off, he had no relapses or side effects under this regimen, with normal lung function and good quality of life.
